# Research on the Physical Properties and Internal Structure of PVP/Nb_2_O_5_ Nanocomposite Coatings

**DOI:** 10.3390/polym17212939

**Published:** 2025-11-03

**Authors:** Paweł Jarka, Pallavi Kumari, Małgorzata Łazarska, Marcin Godzierz, Sonia Kotowicz, Marek Marcisz, Marcelina Bochenek, Łucja Hajduk, Magdalena M. Szindler, Barbara Hajduk

**Affiliations:** 1 Department of Engineering Materials and Biomaterials, Silesian University of Technology, 18a Konarskiego Str., 41-100 Gliwice, Poland; magdalena.szindler@polsl.pl; 2 Centre of Polymer and Carbon Materials, Polish Academy of Sciences, 34 Marie Curie-Skłodowska Str., 41-819 Zabrze, Poland; pkumari@cmpw-pan.pl (P.K.); mgodzierz@cmpw-pan.pl (M.G.); mbochenek@cmpw-pan.pl (M.B.); 3 Faculty of Materials Engineering, Kazimierz Wielki University, 30 Chodkiewicza Street, 85-064 Bydgoszcz, Poland; lazarska@ukw.edu.pl; 4 Institute of Chemistry, University of Silesia, 9 Szkolna Str., 40-006 Katowice, Poland; sonia.kotowicz@us.edu.pl; 5 Faculty of Transport and Aviation Engineering, Silesian University of Technology, 8 Krasińskiego Str., 40-019 Katowice, Poland; marek.marcisz@polsl.pl (M.M.); lh305756@student.polsl.pl (Ł.H.)

**Keywords:** PVP-Nb_2_O_5_ composites, thin films, physical properties, variable temperature spectroscopic ellipsometry

## Abstract

The subject of this study is the effects of various concentrations of niobium pentoxide nanoparticles (Nb_2_O_5_ NPs) on the physical, optical, and thermal properties of thin films of poly(N-vinylpyrrolidone) (PVP). The obtained results indicate that the addition of nanoparticles significantly affects the physical properties of the investigated materials, limiting their optical UV transmittance in the range of 300–500 nm by approximately 20–40% and increasing the material’s resistance to moisture that is present in the surrounding environment. Based on the thermal measurements performed using differential scanning calorimetry (DSC) and variable temperature spectroscopic ellipsometry (VASE), two distinct glass transition temperatures T_g_ for pure PVP and its Nb_2_O_5_ composites were revealed, with an additional intermediate T_g_ appearing in the composites, varying in the range of 135–168 °C (ellipsometric temperature cycle). This intermediate transition indicates the formation of an interfacial region with modified polymer chain mobility due to the interactions occurring between Nb_2_O_5_ nanoparticles and the PVP matrix. The results obtained from the scanning electron microscopy (SEM), Energy Dispersive Spectroscopy (EDS), and detailed Attenuated Total Reflectance-Fourier Transform Infrared Spectroscopy (ATR-FTIR) analyses also confirmed the presence of this interfacial area and indicated that it arises from nanoparticle agglomeration and surface cluster formation. The contact angle measurements revealed that the composites containing 15% and 25% Nb_2_O_5_ exhibited greater hydrophobicity. These results suggest that the investigated composite coatings could be employed as surface coverings to protect against external, environmental influences, such as moisture and UV radiation.

## 1. Introduction

Nanocomposites, in the form of polymer matrices that incorporate organic or inorganic nanoadditives that are uniformly distributed on the nanoscale 10–100 nm and are created through physical mixing processes, present unique chemical and physical characteristics that improve material performance have attracted the attention of researchers around the world [1]. Modern composites reinforced with nanostructures exhibit significantly improved mechanical properties, chemical resistance, thermal stability, reduced permeability, flame resistance, electrical conductivity, and optical performance [2]. The nanofillers used can be, depending on the application, particles, fibers, or even agglomerates embedded in a variety of natural or synthetic polymers. Hence, these materials have great potential for use, especially in aeronautics, automotive, and electronics, as well as in medicine, due to their exceptional qualities [3].

From a production and application perspective, polymer nanocomposites (PNCs) take advantage of the lightweight, flexible nature of polymers, as well as the simplicity of their production and shaping, through cost-effective processes. The PNC materials can be modified depending on the nanoadditive particle’s shape, size, specific surface area, and chemical nature. The selection of nanofiller properties, as well as their homogeneous distribution within the PNC structure, generates phase compatibility, which determines their applicability in various technologies [4,5]. The analysis of scientific literature leads to the conclusion that, depending on the conditions of their synthesis and surface chemistry, the nanoparticles show a strong tendency to form agglomerates. These agglomerates can be classified as hard agglomerates (created by smaller particles connected by sinter necks) and soft agglomerates (created by accumulations of particles connected by attractive physical interactions like van der Waals or hydrogen bridges). The formation of agglomerates is determined by the interactions between nanofiller particles, i.e., their surface chemistry, particle shape, aspect ratio, dimensionality, interparticle distance, and polydispersity [6]. The hard agglomerates can be crushed by high-energy milling, while soft agglomerates are dispersed by shear forces, generating gradients of the mechanical stress.

A particularly interesting application of PNCs is the combination of the functional properties of the matrix polymer and the specific properties of the nanofiller in the structure of a protective coating. The simplicity and cost-effectiveness of producing PNC coatings make them ideal materials for, e.g., UV-blocking applications. Generally, UV light protection coatings are created by incorporating UV-blocking inhibitors or light protection components [5,7]. Currently, UV blockers are used in the form of inorganic fillers, such as metal oxides like TiO_2_, ZnO, SiO_2_, CeO_2_, and Fe_2_O_3_, minerals like CaCO_3_ clays, semiconductors like CdSe, CdS, CdTe, and PbS, and metals and their alloys like Ag, Cu, Au, Fe, and Ge, which are dispersed in polymer matrices [8]. In particular, nanostructured fillers, which achieve a maximum absorption in the UV region (200–400 nm), are capable of efficiently absorbing UV radiation [8,9]. The challenge, however, is to combine the unique optical properties, chemical stability, environmentally friendly nature, and low cost of the inorganic fillers [10] in combination with organic molecules of the matrix. The physical properties of the nanoadditives and their proper distribution within the matrix are crucial for their potential applications [11]. Due to the physical, mechanical, chemical, and thermal properties, polyvinyl alcohol (PVA) [12,13] and polyvinyl pyrrolidone (PVP) are considered to be the matrix for UV protective films. Amorphous PVP is a biocompatible material with high environmental stability [13,14]. However, PVP tends to oxidize when exposed to UV radiation [15,16,17].

It should also be noted that the thermal stability of PNCs in low-cost, large-area protective structures is essential. In polymer film fabrication, scientists aim to increase the crystallinity of films by controlling the evaporation rate or by using external physical force [18,19,20,21,22,23]. The application of external stress (shear, centrifugal force, or capillary forces) contributes to the formation of directional structures in spin-coating, drop-casting, bar-coating, or solution-shear processes.

To date, there has been no detailed study on the effect of one of the most promising metal oxide semiconductors, Nb_2_O_5_, on the photoelectric properties (such as the high refractive index, wide bandgap, good optical quality, and negligible scattering), chemical stability, and thermal stability of PVP/NP structures [16,17].

An extremely important application aspect for polymer-based composite films, especially in applications as protective films, is the determination of their surface wettability, which provides information about surface energy and interactions with liquids. Many well-researched and traditionally used polymers, despite their excellent performance properties, such as their low weight, transparency, and processability, are limited in their use as protective films due to their hydrophilic nature [24]. In such cases, structural modifications of polymers or their combination with hydrophobic polymers are used, e.g., when modifying membranes, which allows obtaining specific surface properties, including hydrophobicity. In the work of Jebali et al., a two-step process was used to obtain hydrophobic PVP films [25]. In the cited work, crosslinking with benzophenone and UV irradiation was used, which led to the formation of water-insoluble PVP, and additionally, the surface of the film was modified by the addition of silica nanoparticles. The related investigation of the hydrophobic properties of polymer/NP coatings was provided by Yousef et al. [26]. The authors modified the PVC/PVP composites using BiVO_4_ nanoparticles, where the addition of these NPs led to a decrease in the water contact angle.

The novelty of this article lies in the use of a unique combination of an organic polymer PVP matrix and inorganic semiconductor nanoparticles Nb_2_O_5_, which allows for the production of coatings with relatively hydrophobic properties that can also protect the coated surface from UV radiation. Such coatings can be relatively easily applied to larger areas, such as parts of various mechanical devices that are exposed to environmental factors. This, in turn, provides a relatively wide range of potential applications, for example, in the automotive, aviation, and medical industries.

## 2. Experimental Section

### 2.1. Materials and Samples Preparation

**Figure 1:**
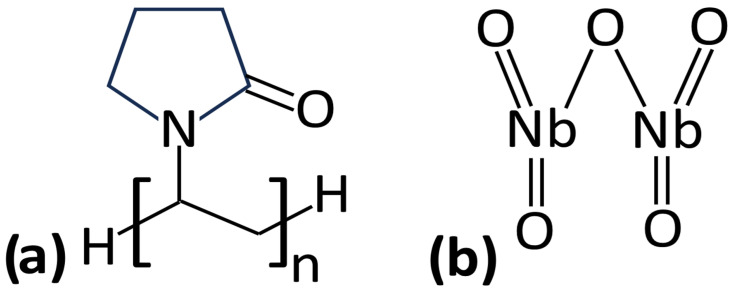
Chemical structures of PVP (**a**) and Nb_2_O_5_ (**b**).

The materials that we used are poly(N-vinylpyrrolidone) (PVP) [27,28] with a molecular weight of 40,000 g/mol, and niobium pentoxide Nb_2_O_5_, with a diameter of around 70 nm (purity 99.9%) [29,30]. PVP and Nb_2_O_5_ were supplied by Sigma-Aldrich. The chemical structures of these materials are presented in Figure 1a,b.

Thin films of PVP and its composites were obtained from a chloroform solution. The weight concentration of each sample solution was constant, i.e., 20 mg/mL. For each sample, 1 mL of a polymer-NP solution was prepared (which ensured the preparation of approximately four films). In Table 1, the percentage indicates the weight fraction of the Nb_2_O_5_ nanoparticles in a 20 mg/mL solution; for example, in samples containing 10% by weight of NPs, 2 mg of Nb_2_O_5_ was used compared to 18 mg of PVP, which constituted the remaining 90%.

Prior to applying the thin films using the spin-coating technique, the solutions were homogenized using the pulse mode with an energy input of 15.1 kJ, power of 20 W, and a pulse time of 11 s for 10 min using a Bandelin Sonopuls homogenizer. The films were coated directly from homogenized solutions with the spinning time t = 60 s, and the spin speed V = 1200 rpm, with the acceleration time t = 2 s.

### 2.2. Methods

To perform the ellipsometric studies, we used the SENTECH SE850E spectroscopic ellipsometer, which was operated by the Spectra Ray 3 software, working within the spectral range of 240 to 2500 nm. Three ellipsometric modes were applied: the transmission mode (using a specific sample holder), the variable angle mode (using a standard automated table), and the variable temperature mode (using a temperature-controlled cell, INSTEC mK1000, operating under decreased pressure). Transmission mode measurements were performed across the whole UV–Vis/NIR spectrum. For incidence angles between 40° and 70°, ellipsometric angles Ψ and Δ were recorded in 5° increments. The protocol described in our earlier studies was followed in the variable temperature experiments [31,32,33,34]. Each sample was heated separately to 250 °C for 5 min while being kept under the pressure of 10^−3^ Torr. The films were then quickly cooled to −100 °C within three minutes. Every temperature cycle was conducted at a heating rate of 2 °C/min. The temperature controller and a liquid nitrogen pump were used to control the temperature of the table (and at the same time, the temperature of the sample).

The transmission mode of the ellipsometer was used for the optical transmission measurements, and the variable angle spectroscopic ellipsometry (VASE) was used for film and roughness thickness measurements and for determining the refractive indices. Using the VTSE, we have determined the thermal transition temperatures.

The DSC analysis was performed on TA DSC 25 Discovery instruments with a heating and cooling rate of 20 °C/min in a constant stream of nitrogen (20 mL/min) atmosphere to 250 °C in the aluminum pans.

SEM imaging, elemental compositional analysis (EDS analysis), and surface roughness measurements were taken using a Phenom XL microscope (Thermo Fisher Scientific) with backscattered electron detection (BSD) that was equipped with an energy-dispersive X-ray spectroscopy (EDS) detector and 3D surface reconstruction module (Phenom 3D). The analysis was conducted at accelerating voltages of 5 kV and 10 kV, after sputter-coating the sample with a gold film to improve surface conductivity.

Contact angle measurements were performed at room temperature by applying a drop of distilled water (with a volume of V = 2–4 µL) onto the test surface using a glass syringe. The contact angle measurement results are the arithmetic mean calculated from 30 images taken at 1 image/sec. Measurements were performed using a CAM101 goniometer (KSV Instruments) equipped with a camera (resolution 640 × 480 pixels) and an external temperature adapter (Intelligent Digital Controller OMRON5EGN). CAM2008 software was used for data analysis and calculations.

X-ray diffraction (XRD) scans were performed on polymer and composite films deposited on cover microscopic glass substrates using a D8 Advance diffractometer (Bruker, Karlsruhe, Germany) with a Cu-Kα cathode (λ = 1.54 Å) in the coupled Two-Theta/Theta mode. The scan rate was 1.2°/min, with a step size of 0.02° in the 2θ range of 2° to 60° (dwell time 1 s). The analysis was performed with DIFFRAC.EVA software V5.1.

ATR-FTIR measurements were provided with a Nicolet 6700 Thermo Fisher device, which worked in the range of 2.5–25 μm.

## 3. Results and Discussion

### 3.1. XRD Analysis

**Figure 2:**
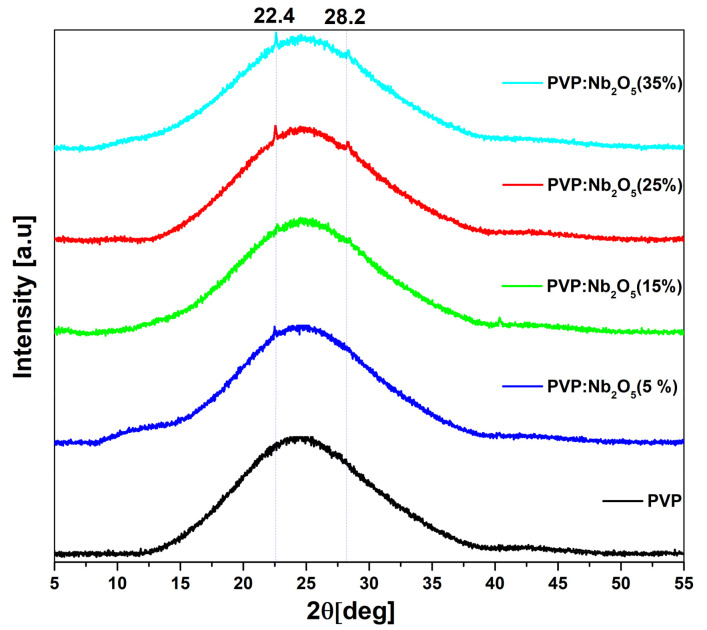
XRD patterns of PVP (black line) and its Nb_2_O_5_ composites (color lines).

The X-ray diffraction (XRD) patterns of the PVP and its Nb_2_O_5_ composite films, with 5, 15, 25, and 35% NPs concentrations, deposited onto microscopic cover glass substrates, are presented in Figure 2.

For reference, the XRD pattern of pure Nb_2_O_5_ nanoparticle powder has been added to the Supplementary Materials (see Figure S1a). There are two visible strong peaks, located at 2θ = 22.4 and 28.2 deg, which are characteristic of this material.

The diffraction spectrum of the pure polyvinylpyrrolidone (PVP) film exhibits a broad, featureless hump, which is characteristic of its amorphous nature. In the case of the sample with a 5% NPs content, we observed one weak peak at 2θ = 22.4°, coming from Nb_2_O_5_, which is related to the low NPs content and formation of sparse NPs clusters. In contrast, the remaining XRD patterns of the composite films, prepared with Nb_2_O_5_, in concentrations of 15, 25, and 35%, displayed two distinct diffraction peaks: −22.4 and 28.2° (the second one also originates from NPs). At the 15% concentration, more agglomerated clusters appear—likely smaller in size. However, due to their higher number, a second peak appeared compared to the 5% sample. This result partially indicates a relatively uniform distribution of nanoparticles in the PVP matrix. At the higher concentrations (25% and 35%), the diffraction peaks become slightly more pronounced, indicating that the Nb_2_O_5_ concentration in these samples is higher and that a larger number of agglomerates have likely formed, possibly with a greater diameter. The XRD pattern of PVP: Nb_2_O_5_ (35%) with a subtracted amorphous hump, with marked Nb_2_O_5_, has been added to the Supplementary Materials (Figure S1b).

### 3.2. Ellipsometric Analysis

**Figure 3:**
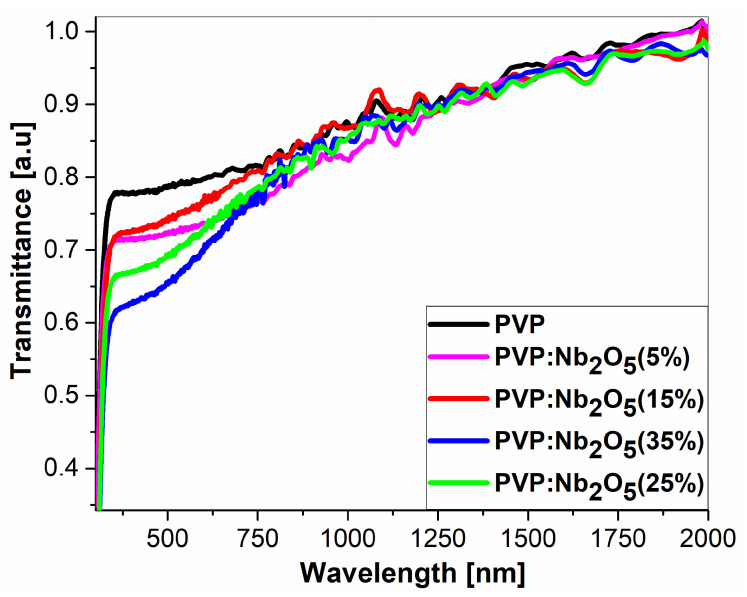
Transmittance of PVP and its Nb_2_O_5_ composites.

The transmission spectra of the pure PVP films and their composites with Nb_2_O_5_ nanoparticles, deposited on a microscopic coverglass, are presented in Figure 3. These measurements were performed using the transmission mode of the ellipsometer. The range of obtained spectra was limited to 2000 nm due to the large spectra fluctuations in the range of 2000–2500 nm.

All the curves were normalized at a wavelength of λ = 2000 nm to account for the differences in the thicknesses of the prepared films (see Table 2). The spectra within the full range and that are non-normalized can be found in Supplementary Materials (see Figure S2).

It can be seen that the spectrum of the pure polymer PVP film is highly transparent, providing light transmission of at least 80% across the entire measured range. In the case of the composite spectra, a significant decrease in the light transmission in the UV–visible range (320–700 nm) can be observed, with the strongest reduction occurring between λ = 320 to 500 nm. These results may be important for potential applications of the investigated materials.

**Figure 4:**
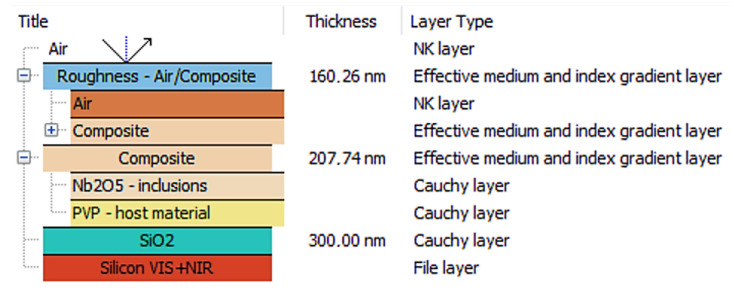
The ellipsometric model used for PVP and its NPs composite spectra fitting.

Variable angle spectroscopic ellipsometry (VASE) was used to determine the refractive index dispersion of the prepared samples. Measurements in the 240–2500 nm wavelength range were performed on PVP polymer films and their Nb_2_O_5_ composites, which were deposited onto silicon substrates coated with 300 nm of SiO_2_. The ellipsometric model, which was used for fitting the theoretical curve to the obtained experimental data (for the Ψ and Δ ellipsometric angles and the degree of polarization), consisted of five component layers: air, a roughness layer, a polymer/composite layer, and two substrate layers, as is presented in Figure 4. A roughness layer was modeled using the effective medium approximation model (EMA), combining the composite layer and the air layer, where air was treated as a non-dispersive medium. The polymer/composite layer was modeled using a Cauchy model for the pure polymer [35,36,37], while the composite was modeled using an EMA-type model, fitted for PVP and a Cauchy model of Nb_2_O_5_ material layer, taken from the database of ellipsometric models that are available in SpectraRay 3 software. Finally, an air layer with a refractive index of 1 was included. The so-called volume fraction coefficient was used to determine the Nb_2_O_5_ nanoparticles content in the PVP matrix. This coefficient corresponds to the volume fraction of nanoparticles relative to the volume of polymer and is not identical to the weight percentage ratio of nanoparticles to polymer. The formula for the volume fraction coefficient *f* is given in Equation (1), and the obtained values are presented in Table 2.

The Bruggeman formula yielded the volume fraction coefficient [38] that was used for a polymer or composite layer:

(1) $$f\frac{\varepsilon_{i}-\varepsilon_{eff}}{\varepsilon_{i}+{2\varepsilon }_{eff}}+\left(\;1-f\right)\frac{\varepsilon_{m}-\varepsilon_{eff}}{\varepsilon_{m}+{2\varepsilon }_{eff}}=0$$

where *f* is the volume fraction coefficient of nanoparticles, *ε_i_* is the nanoparticles’ dielectric coefficient (inclusion material), *ε_m_* is the polymer dielectric coefficient (host material), and *ε_ff_* is an effective dielectric coefficient of the investigated material (composite). Based on this formula, it was possible to derive the dependence relation on the coefficient of volumetric fraction:

(2) $$f\approx \frac{\varepsilon_{eff}-\varepsilon_{m}}{{3\varepsilon }_{m}}\cdot \frac{\varepsilon_{i}+2\varepsilon_{m}}{\varepsilon_{i}-\varepsilon_{m}}$$

**Figure 5:**
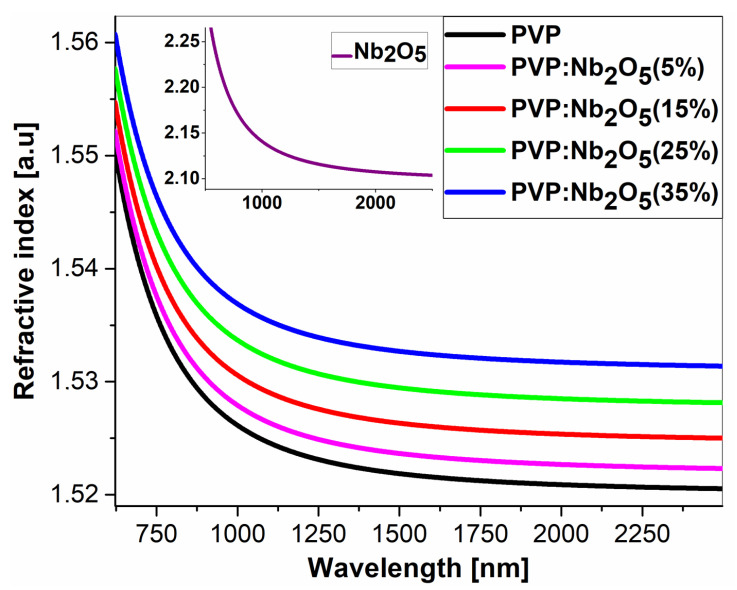
Values of refractive indices of PVP and its composites with Nb_2_O_5_. The refractive index of Nb_2_O_5_ has been added as the inset for comparison.

The obtained volume fraction coefficient values, along with the film thickness, surface roughness, and refractive index for the wavelength λ = 2000 nm, are shown in Table 2. Also, the dispersions of refractive indices, generated for PVP, Nb_2_O_5_ (Sentech ellipsometric database), and for composites with different Nb_2_O_5_ percentage contents that were deposited onto silicon substrates, are presented in Figure 5. It is noticeable that the dispersions are presented in the range of 500 to 2500 nm. The reason for this approach is the degree of light polarization in the 240–500 nm region, the deviation of which significantly exceeds 10%. Additionally, the number of spectra used in the theoretical fit was limited to incidence angles of 40–50°, because for angles of 60–70°, the degree of polarization significantly deviated from 1 over the entire measured wavelength range. The refractive index *n* of pure PVP is around 1.521, while that *n* of Nb_2_O_5_ is approximately 2.097. With respect to the weight percentage of the nanoparticles, the *n* value for Nb_2_O_5_ should significantly affect the refractive index of the composite. However, ellipsometric fits indicate otherwise. The *n* values for the individual NPs percentages—5%, 15%, 25%, and 35%—are 1.522, 1.525, 1.528, and 1.531, respectively, and thus do not differ significantly from the refractive index of the polymer matrix. This is due to the low values of the volume fraction coefficients, which for the composites are f = 0.004, 0.010, 0.017, and 0.024, respectively. Note that *f* is the factor that is determined from the optical model with the best possible fit (for the lowest mean square error (MSE) value obtained). Being dimensionless, the volume factor differs from the weight factor. Because the *f* values are low, this means that only a small percentage of nanoparticles contained in the polymer matrix have an active influence on the composite’s refractive index. Another reason for such low *f* values may be the relatively high surface roughness, which is reflected in the determined surface roughness coefficient values (see Table 2). The obtained ellipsometric results are in very good agreement with the SEM surface roughness analysis (see Supplementary Materials, Figure S2).

Based on the obtained results, we can assume that the nanoparticles contained in the polymer form agglomerates that cause light scattering in the 300–500 nm range. However, this has no significant effect on light transmittance in the remaining spectral range, where the wavelength is longer, and, therefore, the nanoparticle agglomerates become “invisible to the beam.” The low refractive index values measured at a wavelength of 2000 nm also confirm this fact. The formation of agglomerates is confirmed by the results obtained using SEM microscopy and may also affect the thermal properties of the resulting composite films.

### 3.3. Thermal Analysis

**Figure 6:**
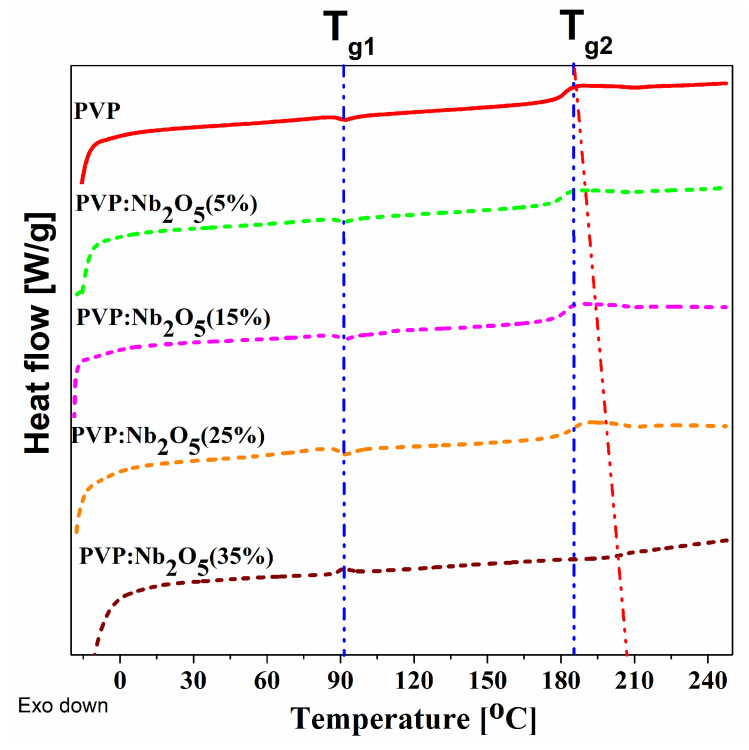
The DSC plots, obtained with a heating rate of 20 °C/min for pure PVP and its Nb_2_O_5_ composites (5, 15, 25, and 35%). The red line represents the T_g_ deviation for the highest nanoparticle concentration compared to pure PVP.

The thermal analyses of pure PVP and its composites were performed using two methods: DSC and VTSE, and the obtained results were then compared. The DSC analysis included measurements of the starting materials in powder form after solvent evaporation. The obtained DSC plots for the PVP and composites are presented in Figure 6, and the individual, detected thermal transition temperatures are shown in Table 3. The results indicate the presence of two glass transition temperatures (T_g_). For both pure PVP and its composites, the first temperature (T_g1_) is equal to 88–89 °C and is, therefore, practically constant. All of the results also indicate a second glass transition temperature (T_g2_), with values detected for pure PVP and its composites with Nb_2_O_5_ (5, 15, 25, and 35%) of 188, 181, 180, 183, and 204 °C, respectively. The typical T_g_ temperature of PVP is included within the temperature range of 150–180 °C [39,40,41,42]. In the case of the plot, obtained for the pure material, we have recorded the temperatures of 88 and 188 °C. Two explanations for the obtained results are possible. The first approach suggests that T_g1_ = 88 °C is a temperature derived from secondary β-segmental relaxation, which was very well described by Vyazovkin and Dranc [43]. The pyrrolidone ring’s rocking motions as it rotates around the C-N bond are the cause of this phenomenon, which is independent of the molecular weight. Compared to the glass transition energy of the polymer backbone, this type of motion has a lower energy barrier.

The second possible explanation is the presence of a PVP fraction with a very low molar mass, which could indicate that the investigated material is a mixture of two molecular weight fractions (40,000 g/mol and a much lower one). Using the Flory–Fox relation (Equation (3)) for the amorphous polymers [44], the molar mass of the second fraction can be determined to be approximately 3000 g/mol. The Flory–Fox relation is described with the following well known equation:

(3) $$T_{g}=T_{g\infty }-\frac{C}{M}$$

where $T_{g\infty }$ is the glass transition of the polymer with a high molecular mass (in our case, it is around 180 °C), *M* is the average molecular mass, and *C* is a constant characteristic of the individual polymer. In the case of the second *T_g_*_2_ temperature, the largest shift is observed for the highest nanoparticle concentration. Since this *T_g_*_2_ value is typical for PVP, the changes in its value can be explained by the presence of nanoparticles. Nb_2_O_5_ nanoparticles, present in the polymer matrix, reduce the vibrations of the polymer backbone, stiffening it. This interaction increases the energy barrier, beyond which the glass transitions can occur.

**Figure 7:**
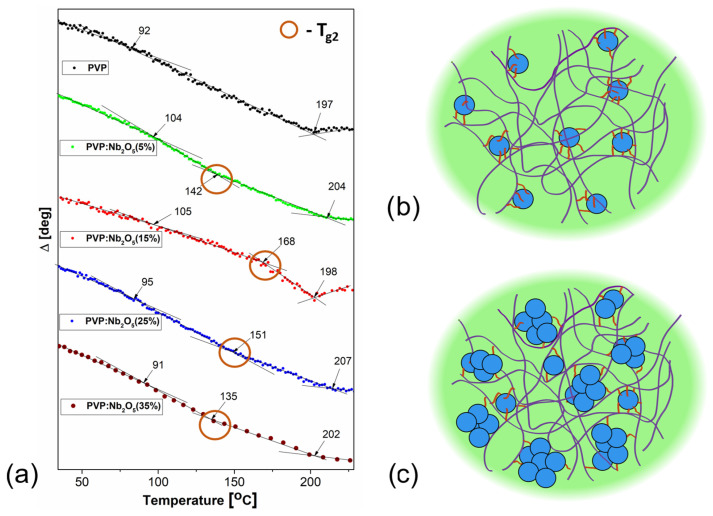
The ellipsometric angle Δ plot at 900 nm as a function of temperature for PVP and its Nb_2_O_5_ composites, (**a**) and nanoparticles distributed at lower (**b**) and higher concentrations (**c**).

Referring to our previous studies of thermal transitions of composite films [45,46] using variable temperature spectroscopic ellipsometry, it should be noted that it is quite convenient and quick to use raw ellipsometric data for a selected wavelength [47,48,49]. In this paper, we present thermal studies that were conducted using differential scanning calorimetry methods and compare them with the results obtained using the VTSE method. Here, we have selected the Ψ angle at a wavelength of λ = 900 nm. The restricted spectral range used for temperature monitoring and the high transmission in this range are the reasons for selecting this wavelength. The ellipsometric temperature cycles, recorded for films of PVP and its composites, deposited onto silicon substrates, are presented in Figure 7, and the obtained temperatures are presented in Table 3. For pure PVP, we have detected two temperature values: 92 and 197 °C, which correspond to the two *T_g_* values obtained using the DSC method. This is typical, as the differences between the DSC and VTSE scans usually range from 10 to several degrees Celsius due to the different physical forms of the investigated materials (film in the case of VTSE; powder in the case of VTSE) [32]. Observations of the ellipsometric temperature scans led to very interesting conclusions. For the composites, whose content of Nb_2_O_5_ nanoparticles is equal to 5 and 15%, we noticed an increase in the first T_gV1_ value, from 92 °C (recorded for pure PVP film) to 104 and 105 °C, respectively. For the concentrations of 25 and 35%, the temperature values were shifted again to values closer to those for pure PVP: 95 and 91 °C, respectively. For T_gV3_, which corresponds to the T_g2_ (the second glass transition temperature that is observed in DSC plot), we observed shifts in the temperature range of 198–207 °C. It can be easily noticed that, unlike the results obtained using the DSC method for the powdered material, each ellipsometric curve shows an intermediate temperature, which is absent in the case of the PVP matrix film without the addition of nanoparticles. The value of this intermediate temperature T_gV2_ changes varies significantly with the NP concentration, being the highest for a concentration of 15% Nb_2_O_5_, and the lowest for 35%. Changes in T_gV1_ and T_gV3_ can be easily explained by the stiffening of the PVP polymer chains. The most important temperature appears to be the T_gV2_ (Figure 7a), which can be related to the nanoparticles’ agglomeration. Because spectroscopic ellipsometry is an extremely sensitive method for detecting changes in the physical parameters (e.g., the thickness), the effect must be linked to the interaction between the Nb_2_O_5_ NPs and the polymer. In the work of Ali et al. [50], it was shown that C=O···HO–Nb hydrogen bonds formed between Nb_2_O_5_ nanoparticles and the PVP/PVA polymer matrix, where C=O originates from the PVP pyrrolidone ring, the -OH group from PVA, and Nb-OH represents hydroxyl groups on the niobium pentoxide surface, which can act as donors or acceptors of hydrogen bonds. We believe that a similar problem occurs in our composite films. When nanoparticle concentrations are lower and more uniformly distributed in the polymer matrix, larger numbers of such linkages can be formed, resulting in a larger amount of this “third”, intermediate phase. The glass transition temperature T_gV2_ appears as a consequence of the increased interfacial surface area, formed between the Nb_2_O_5_ nanoparticles and the PVP polymer, leading to the formation of a PVP phase with modified chain mobility (Figure 7b). At higher nanoparticle concentrations (25% and 35%), a large number of nanoparticle agglomerates are present, causing the T_gV2_ temperature to be shifted to 151 °C and 135 °C, respectively. Fewer hydrogen bonds develop around larger agglomerates, which pushes the polymer chains apart (Figure 7c). Furthermore, the so-called dilution effect applies here: when the weight concentration of the solutions from which the films were derived remains constant, the sample contains less polymeric material and proportionally more nanoparticles than samples with lower concentrations. These two factors lead to a decrease in T_gV2_ at the highest Nb_2_O_5_ concentrations. It should be noted that T_gV2_ does not appear in the DSC results because the DSC results were presented for the same material in powder form. This temperature is related to the agglomeration of nanoparticles in the polymer matrix when an interface forms between PVP and Nb_2_O_5_ in layers where the material is continuous because it has been exposed to a solvent.

### 3.4. ATR-FTIR Analysis

**Figure 8:**
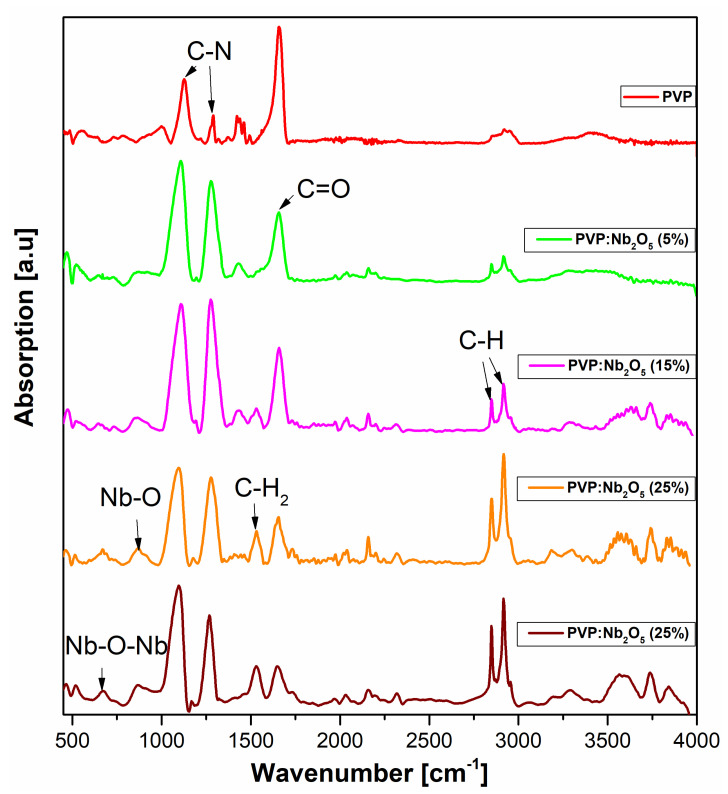
The ATR-FTIR spectra of PVP and its Nb_2_O_5_ composites.

Unannealed films of PVP and its Nb_2_O_5_ composites, deposited on silicon substrates, underwent FTIR investigation. The clean substrate spectrum was subtracted from all spectra, and the baseline was applied to each of them. For clarity, Figure 8 displays the spectra within the 450–4000 cm^−1^ wavenumber range. The spectra of individual samples, highlighting the most significant peaks, are displayed in the Supplementary Materials in Figure S3a–e.

Peaks at frequencies of 734, 781, 1004, 1130, 1288, 1423, 1660, 2856, 2920, and 2954 cm^−1^ are present in the PVP spectrum. The PVP spectrum (Supplementary Materials Figure S3a) features peaks at frequencies of 734, 781, 1004, 1130, 1288, 1423, 1660, 2856, 2920, and 2954 cm^−1^. Of these, 734 is a peak originating from C-H vibrations in the pyrrolidone ring, and 781 is a deformational C-H vibration. The peaks placed at about 1130 and 1288 cm^−1^ originate from C-N vibrations, where the first is the C-N stretching vibration, associated with C-C stretching within the pyrrolidone ring, and the second one (1288) is a C–N stretching vibration in the N–C=O, characteristic for the pyrrolidone group. The 1423 and 1454 peaks are coming from CH2 scissor vibrations, 1660 from valence vibrations of the C=O group, and vibrations 2856, 2920, and 2954 originate from the C-H groups. The 2856 peak originates from asymmetric vibrations of C-H in CH_2_ groups in the polymer chain, the 2920 is the asymmetric vibration of the same C-H bonds, and the 2952 peak is the asymmetric vibration of C-H in CH_3_ in the pyrrolidone ring or in the polymer chain endings.

In the spectrum of PVP with a 5% Nb_2_O_5_ content (Supplementary Materials Figure S3b), the peaks are located at wavenumber values of 645, 881, 1108, 1280, 1430, 1657, 2161, 2849, and 2913 cm^−1^. Two new peaks can be observed, located at frequencies of 645 and 881 cm^−1^. These peaks originate from the Nb-O-Nb bridge vibrations and the Nb-O stretching vibrations, respectively. The presence of Nb_2_O_5_ NPs was also fully confirmed by EDS analysis (see Supplementary Materials, Section S4). The additional peak appearing at the concentration of 15% of NPs at about 1538 cm^−1^ comes from coupled N–C=O/C–N vibrations in the pyrrolidone ring. The origination of individual peaks in the rest of the spectra, with the NPs concentration 15–35% (Supplementary Materials Figure S3c–e), is the same.

In the subsequent composite spectra, we can observe that the intensity of C-N peaks (1130 and 1288 cm^−1^) is noticeably higher compared to the spectrum of pure PVP. It is the result of the interaction of pyrrolidone rings with Nb_2_O_5_, which influences the dipole moments of the C–N and N–C=O vibrations. We observed the highest intensity peak of 1288 for the PVP spectrum with 15% NPs.

The decreasing increase in the CH_2_ peak’s intensity (1438) at the concentrations 25 and 35%, and the appearance of the new one at 1538 cm^−1^ at the 15% NP concentration, means that until “this moment”, exposed CH_2_ groups for the higher NPs concentrations exist. The presence of both peaks indicates the optimum interfacial PVP-Nb area. For the concentrations 25–35%, where we can find the agglomeration effects, the CH_2_ groups are “locked” inside the agglomerate, while the C–N and N–C=O groups are still interacting with the Nb_2_O_5_ surface.

The peak originating from the C=O group (1656) decreases with the NP concentration, and the intensity of the C-H peaks (2850–2952) noticeably increases with the NP concentration. These results clearly confirm the formation of C=O..HO-Nb groups. The formation of such bonds partially limits the rotational vibrations of the C=O group. The more that these groups are involved, the NPs partially organize the polymer chains, simultaneously stiffening the entire matrix. As a result of the partial ordering, a large number of dipoles forming the C-H groups align in parallel, resulting in a stronger signal in the infrared spectrum due to their combined dipole moment. The bands originating from the C-H groups remain strong at a concentration of 35%, while the sample’s hydrophobicity decreases. This indicates that despite the chains being ordered at the molecular level, the macroscopic surface becomes more inorganic and dominated by Nb_2_O_5_, which reduces the composite’s hydrophobic properties, which is what was confirmed in further contact angle analyses.

### 3.5. Microscopic and Contact Angle Analyses

**Figure 9:**
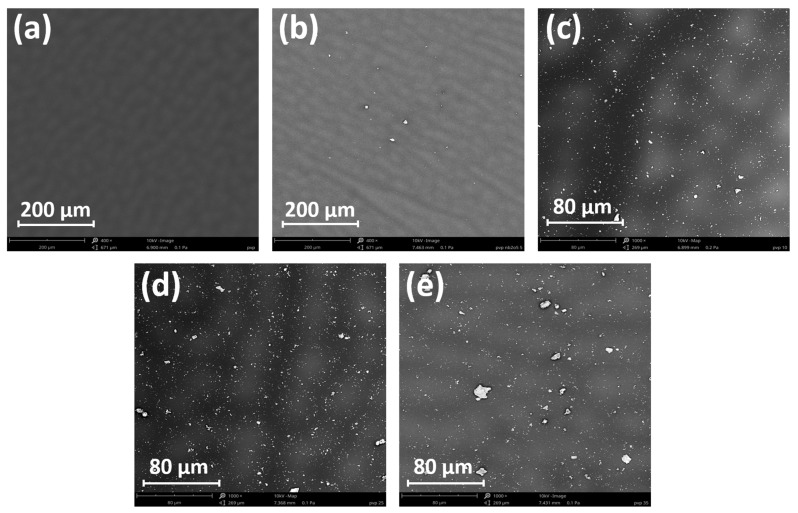
SEM pictures of the morphology of PVP (**a**) and its Nb_2_O_5_ composites at concentrations of 5% (**b**), 15% (**c**), 25% (**d**), and 35% (**e**).

The pieces of the same PVP and its composites with Nb_2_O_5_ samples were tested using SEM. Figure 9a–e presents morphology images at magnifications of 400× (a–b) and 1000× (c–e). Nb_2_O_5_ nanoparticles are visible on the surface of the images as bright spots. Figure 9a shows the surface of a pure PVP matrix without the addition of nanoparticles, and Figure 9b presents the morphology of the PVP film with a 5% Nb_2_O_5_ content. A few nanoparticle agglomerates are visible in the field of view. Figure 9c–e, which correspond to concentrations of 15, 25, and 35% Nb_2_O_5_ content, show larger and more numerous nanoparticle agglomerates. Based on the obtained results, we can conclude that the PVP/Nb_2_O_5_ films reveal significant morphological changes compared to the materials with a lower NPs concentration. The polymer matrix shows pronounced heterogeneity, and the presence of numerous highly contrasting bright regions indicates well-dispersed Nb_2_O_5_ nanoparticles. The higher content of the inorganic phase promotes the formation of particle clusters, suggesting progressive agglomeration. The increased number and size of the bright regions reflect the intensified influence of Nb_2_O_5_ on the composite’s microstructure. SEM images of the Nb_2_O_5_ cluster, with the diameter marked, are shown in Supplementary Materials in Figure S4c.

**Figure 10:**
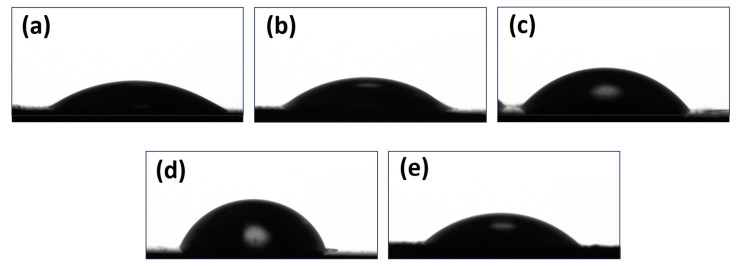
Measurements of the contact angle of PVP (**a**) and its Nb_2_O_5_ composites at concentrations of 5% (**b**), 15% (**c**), 25% (**d**), and 35% (**e**).

The obtained contact angle measurement results (Figure 10) are consistent with the previously obtained results. In Figure 10a–d, the hydrophobicity of the films increases with the NPs concentration. In the case of the pure PVP film, the surface is smooth and moderately hydrophilic. For a concentration of 5% of Nb_2_O_5_, the hydrophilicity decreases slightly, with the appearance of isolated nanoparticle clusters when compared to the pure material. Hydrophobicity increases gradually up to a concentration of 25% and then decreases sharply when it reaches 35% (Figure 10e). This phenomenon can be explained by the supersaturation of the polymer matrix with the higher concentration of Nb_2_O_5_ NPs. Larger and more numerous agglomerates are formed; thus, the sample surface becomes more textured, leading to a greater exposure of hydrophilic Nb_2_O_5_ regions. Furthermore, the increased number of nanoparticle aggregates enhances the film’s porosity and capillarity, which, in turn, raises the overall hydrophilicity of the sample.

## 4. Conclusions

In this paper, we investigated PVP and its composites with Nb_2_O_5_ nanoparticle thin films. This study focused on comparing the physical properties of the samples with varying nanoparticle concentrations.

XRD analysis has confirmed the amorphous structure of the PVP matrix and the presence of Nb_2_O_5_ NPs in the polymer matrix, which was also confirmed using EDS analysis.

The transmission spectra revealed an increased UV absorption in the 320–700 nm range, with a higher nanoparticle content associated with a greater reduction in optical transmittance in this region, while the remainder of the spectra range remained transparent. According to the VASE experiments, the active contribution to the changes in the *n* dispersion was minimal (1.521–1.531 at λ = 2000 nm). The low values of the volume fraction coefficient clearly indicate that only a small proportion of Nb_2_O_5_ NPs actively contribute to the refractive index, confirming the presence of particles and agglomerates on the surface of the composite. The thermal analyses (DSC and VTSE) clearly showed the presence of two glass transition temperatures, both in pure PVP and in its composites. In the case of powdered material, T_g1_ remained constant and originated from the pyrrolidone moiety, and a variable T_g2_ was attributed to nanoparticle-induced stiffening of the polymer chains. The VTSE studies revealed an additional glass transition temperature (T_gV2_), indicating the formation of an interfacial region between the PVP matrix and Nb_2_O_5_ nanoparticles due to hydrogen bonding at their interface. Due to the varying number of such bonds, we associate the changes in the T_g2_ temperature with an increase in Nb_2_O_5_ agglomerations. These results were confirmed by ATR-FTIR analysis, which clearly indicated the formation of C=O..HO-Nb groups. The SEM results revealed a homogeneous distribution of nanoparticles within the matrix, as well as an increase in the number and size of agglomerates. The contact angle measurements suggest that the most suitable concentrations appear to be 15% and 25%, at which the composite becomes relatively hydrophobic.

The results of this study demonstrate that the investigated PVP/Nb_2_O_5_ composites can be employed as prospective coatings to protect surfaces from UV radiation while maintaining the coating’s relative hydrophobicity.

## Figures and Tables

**Table 1:** Percentage content and weight of individual chloroform/polymer/NP solutions.

**Polymer content (%)**	100	90	85	75	65
**NPs content (%)**	0	10	15	25	35
**PVP weight (mg)**	20	18	17	15	13
**Nb_2_O_5_ Weight (mg)**	0	2	3	5	7

**Table 2:** Physical parameters of PVP and its composites.

Thin Film	PVP	PVP: Nb_2_O_5_ (5%)	PVP: Nb_2_O_5_ (15%)	PVP: Nb_2_O_5_ (25%)	PVP: Nb_2_O_5_ (35%)
**Refractive index *n* [a.u]** (for λ = 2500 nm)	1.521	1.522	1.525	1.528	1.531
**Fraction coefficient** **f [a.u]**	0	0.004	0.010	0.017	0.024
**Thickness of films on SiO_2_** **d [nm]**	303	371	255	466	208
**Roughness of films on SiO_2_** **r [nm]**	146	134	128	162	160
**Thickness of films on micr. cover glass** **d [nm]**	257	302	270	285	340
**Roughness of films on micr. cover glass** **r [nm]**	101	120	97	115	134

**Table 3:** Thermal properties: Glass transition temperature of polyvinylpyrrolidone and its composites with Nb_2_O_5_ using DSC and VTSE.

	DSC (Powder)		Temperature Ellipsometry (Films)		
Sample	T_g1_ (°C)	T_g2_ (°C)	T_gV1_ (°C)	T_gV2_ (°C)	T_gV3_ (°C)
PVP	88	188	92	-	197
PVP: Nb_2_O_5_ (5%)	88	181	104	142	204
PVP: Nb_2_O_5_ (15%)	88	180	105	168	198
PVP: Nb_2_O_5_ (25%)	89	183	95	151	207
PVP: Nb_2_O_5_ (35%)	88	204	91	135	202

## Data Availability

The original contributions presented in this study are included in the article. Further inquiries can be directed to the corresponding authors.

## Supplementary Materials

The following supporting information can be downloaded at: https://www.mdpi.com/article/10.3390/polym17212939/s1, Figure S1: XRD pattern of Nb2O5 nanoparticles (a), pattern of PVP: Nb2O5 composite, with subtracted amorphous PVP hump (b), Figure S2: Non-normalised transmission spectra, Figure S3: FTIR spectrum of PVP (a), FTIR spectrum of PVP: Nb_2_O_5_ (5%) (b), FTIR spectrum of PVP: Nb_2_O_5_ (15%) (c), FTIR spectrum of PVP: Nb_2_O_5_ (25%) (d), FTIR spectrum of PVP: Nb_2_O_5_ (35%) (e), Figure S4: PVP EDS analysis (a), PVP: Nb_2_O_5_ EDS analysis (b), 7000× zoom on Nb_2_O_5_ cluster (c), Figure S5: SEM roughness 3D and 2D profile of PVP (a), SEM roughness 3D and 2D profile of PVP: Nb_2_O_5_ (5%) (b), SEM roughness 3D and 2D profile of PVP: Nb_2_O_5_ (15%) (c), SEM roughness 3D and 2D profile of PVP: Nb_2_O_5_ (25%) (d), SEM roughness 3D and 2D profile of PVP: Nb_2_O_5_ (35%) (e), Cut out of PVP: Nb_2_O_5_ map (f), Tables S1 and S2: EDS spot of PVP, Table S3: EDS spot of Nb_2_O_5_, Table S4: Results of roughness SEM analysis.
