# Supplementary material for: Research on the Physical Properties and Internal Structure of PVP/Nb2O5 Nanocomposite Coatings
Source: Polymers (Basel). 2025 Nov 3;17(21):2939. doi: 10.3390/polym17212939 (PMC12608762; doi:10.3390/polym17212939)
Supplement: Supplementary file 1 [file polymers-17-02939-s001.zip › polymers-3916834-supplementary.pdf]

Supplementary Materials for the article:

Research on the Physical Properties and Internal Structure of PVP/Nb_2O_5 Nanocomposite Coatings

Paweł Jarka [1,*], Pallavi Kumari [2], Małgorzata Łazarska [3], Marcin Godzierz [2], Sonia Kotowicz [4], Marek Marcisz [5], Marcelina Bochenek [2] , Łucja Hajduk [5], Magdalena M. Szindler [1] and Barbara Hajduk [2,*]

[1] Department of Engineering Materials and Biomaterials, Silesian University of Technology, 18a Konarskiego str., 41-100 Gliwice, Poland
[2] Centre of Polymer and Carbon Materials, Polish Academy of Sciences, 34 Marie Curie-Skłodowska str.,
41-819 Zabrze, Poland
[3] Faculty of Materials Engineering, Kazimierz Wielki University, 30 Chodkiewicza Street, 85-064 Bydgoszcz, Poland
[4] Institute of Chemistry, University of Silesia, 9 Szkolna Str., Katowice 40-006, Poland
[5] Faculty of Transport and Aviation Engineering, Silesian University of Technology, Krasińskiego 8,
40-019 Katowice, Poland
* Correspondence: pawel.jarka@polsl.pl (P.J.); bhajduk@cmpw-pan.pl (BH.)

1S. XRD analysis

Here we present the XRD pattern of Nb_2O_5 nanoparticles (a) and pattern of PVP:Nb_2O_5 composite, with subtracted amorphous PVP hump (b).

a)

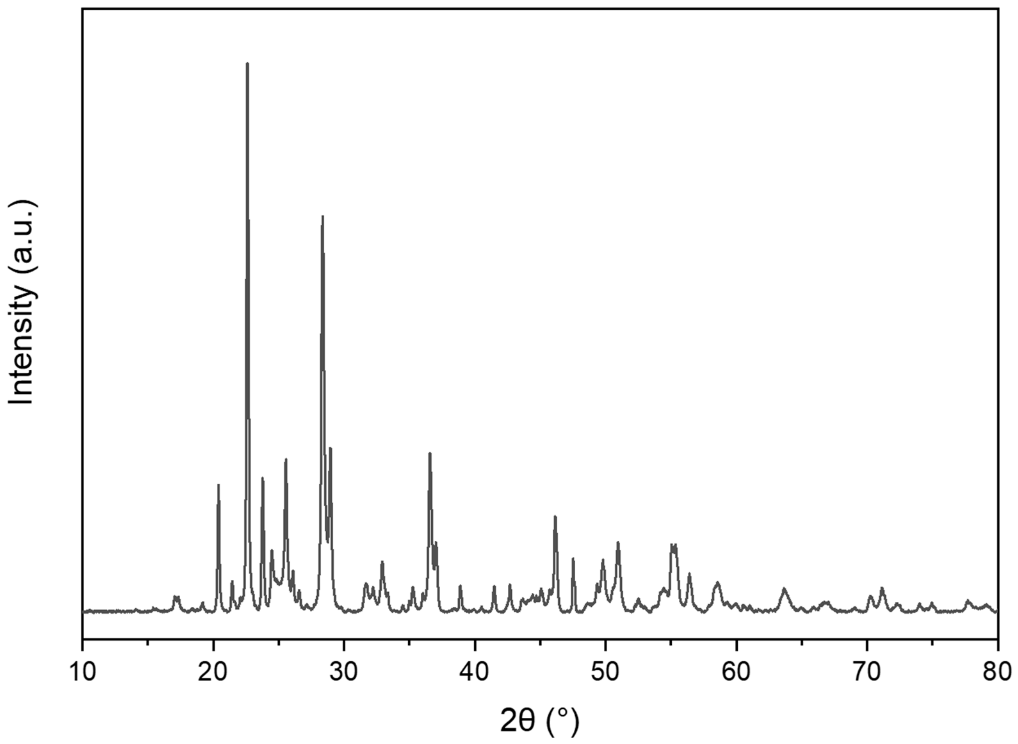

b)

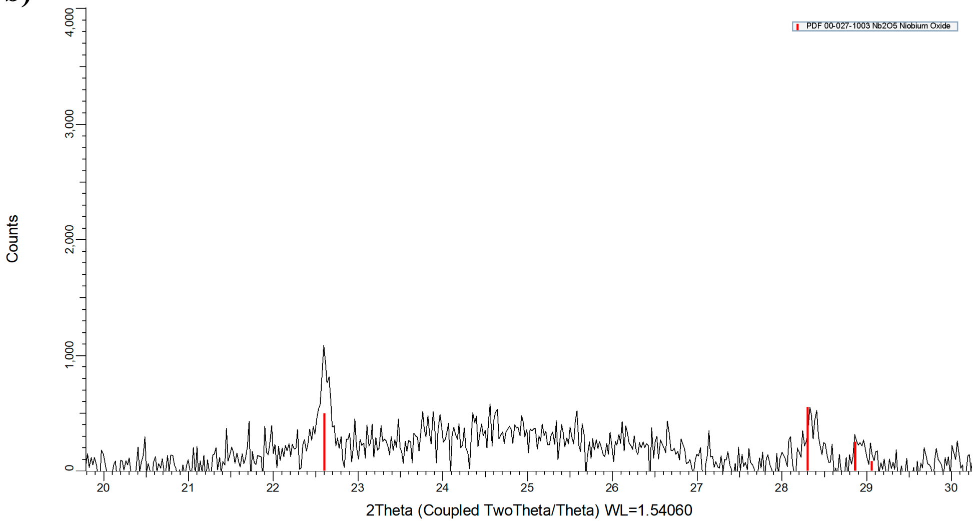

2S. UV-VIS transmission

Below we present non-normalised spectra, taken in the full of wavelength range.

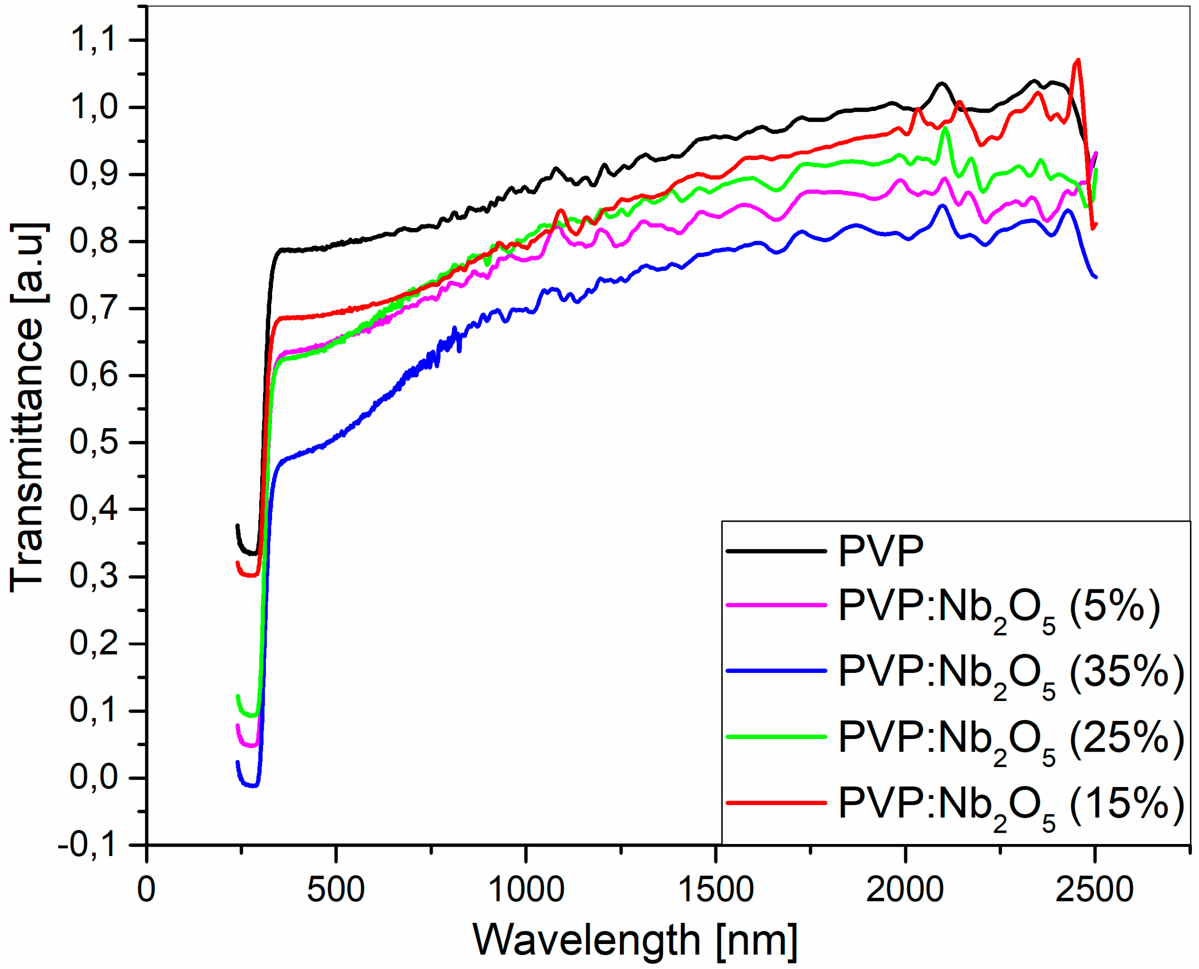

3S. ATR-FTIR analysis:

The FTIR absorption spectra were taken on PVP and its Nb_2O_5 NPs composites deposited onto silicon substrates. The spectrum of PVP is presented in Fig. 3S a). The rest of spectra for 5, 15, 25 and 35% concentrations are presented in Figs 3S b)-e), respectively. The peaks in composite spectra, characteristic coming from the Nb-O-Nb bridge vibrations and the Nb-O stretching vibrations are placed at about 645 and 881 cm^{-1} [1s-3s].

a)

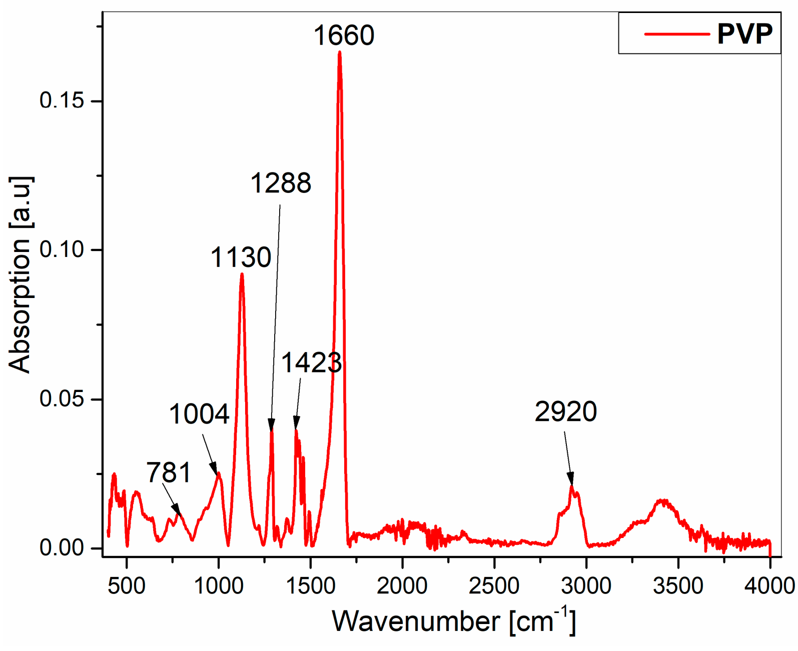

b)

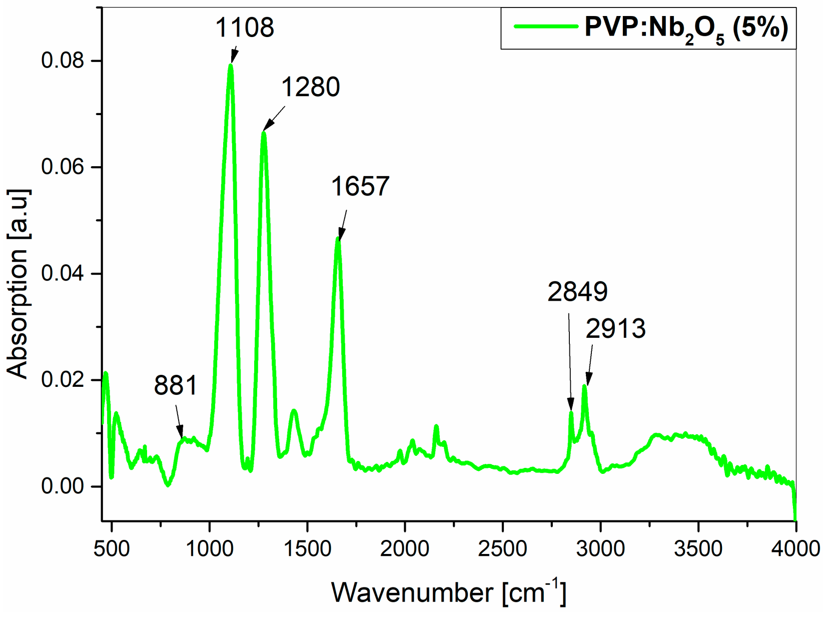

c)

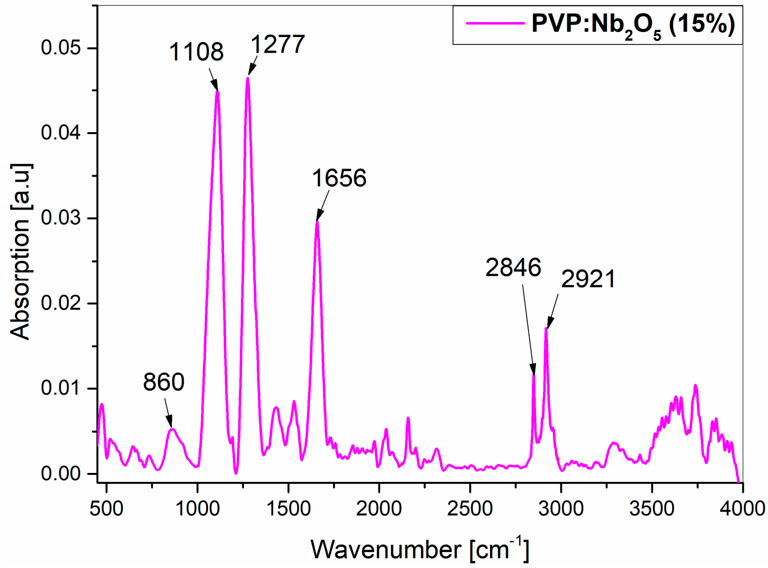
PVP:Nb_2O_5 (15%)
Absorption [a.u]
Wavenumber [cm^{-1}]
1108
1277
1656
2846
2921
860
0.00
0.01
0.02
0.03
0.04
0.05
500
1000
1500
2000
2500
3000
3500
4000

d)

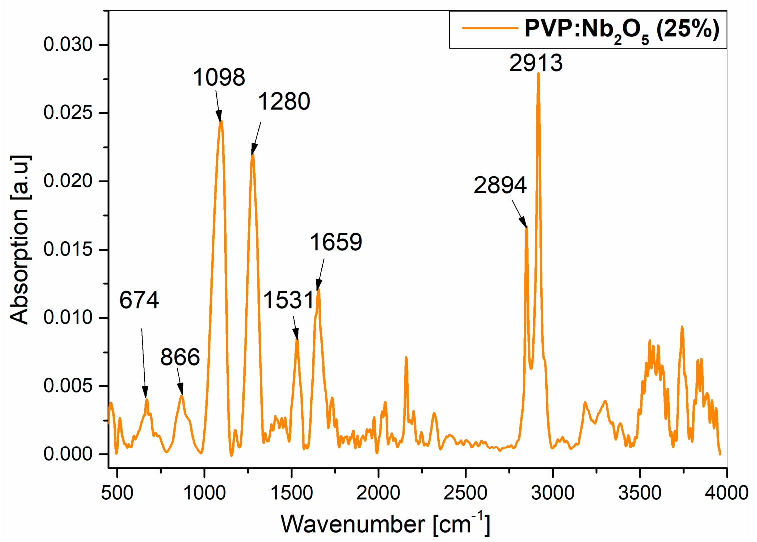
PVP:Nb_2O_5 (25%)
Absorption [a.u]
Wavenumber [cm^{-1}]
1098
1280
2913
2894
1659
1531
674
866
0.000
0.005
0.010
0.015
0.020
0.025
0.030
500
1000
1500
2000
2500
3000
3500
4000

e)

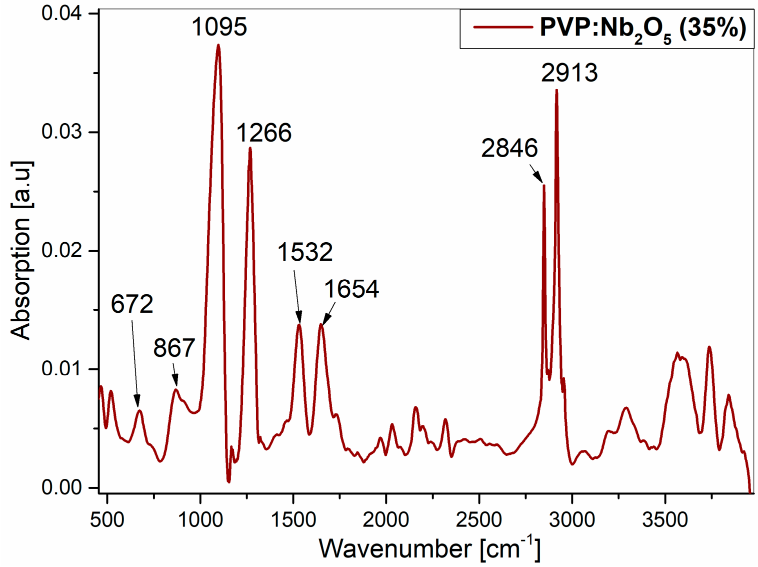
PVP:Nb_2O_5 (35%)
Absorption [a.u]
Wavenumber [cm^{-1}]
1095
1266
2913
2846
1532
1654
672
867
0.00
0.01
0.02
0.03
0.04
500
1000
1500
2000
2500
3000
3500

4S. EDS analysis results:

The samples were coated with a thin layer of gold (Au) using the vacuum sputtering method to reduce surface charging effects during the analysis. Measurements were carried out under high vacuum conditions with an accelerating voltage of 5 kV, which provided good resolution for signals from light elements (C, N, O) present in the polymer matrix. The analyses were performed in two modes: spot analysis, to determine the local chemical composition at selected points on the surface of the pure PVP sample, and elemental mapping, to determine the elemental distribution in the PVP/Nb_2O_5 composite sample. Several independent analyses were conducted for each sample, including both EDS spectra acquisition and elemental distribution mapping (C, O, N, Si, Nb, Au). Elemental identification and quantification were carried out using ProSuite software (Thermo Fisher), applying a semi-quantitative method with ZAF correction (atomic number–absorption–fluorescence). The obtained results are reported in atomic percent (at%). The presence of niobium (Nb) in the PVP/Nb_2O_5 composite sample was confirmed both in the EDS spectra and in the elemental distribution maps. The presence of gold (Au) observed in the analyses resulted from the sputtered coating applied during sample preparation and is not a component of the material.

In the case of pure PVP, the EDS spectra were dominated by signals from carbon (C), oxygen (O), and nitrogen (N), which is consistent with the typical chemical composition of polyvinylpyrrolidone. Small amounts of silicon (Si) were also observed in the samples, originating from the substrate. Additionally, a gold (Au) signal appeared in the spectra, resulting from the sputtered coating applied to the sample surface to ensure electrical conductivity.

In the analysis of the PVP/Nb_2O_5 composite, in addition to the elements originating from the polymer matrix, a clear presence of niobium (Nb) was detected, which unequivocally confirms the incorporation of niobium oxide nanoparticles into the polymer structure. The average niobium content in the analyzed mapping area was approximately 5.1 at%, accompanied by an increase in oxygen content (about 24.4 at%) and a decrease in carbon content (about 43 at%) compared to the pure PVP sample. The change in the C/O ratio indicates the presence of an inorganic oxide phase and confirms the contribution of Nb_2O_5 to the composite. Elemental distribution maps revealed a uniform distribution of Nb and O within the polymer matrix, suggesting relatively good dispersion of Nb_2O_5 nanoparticles throughout the PVP structure.

a) PVP

Table 1. Spot

Element Number	Element Symbol	Element Name	Atomic Conc.
6	C	Carbon	56.31
8	O	Oxygen	28.26
7	N	Nitrogen	13.03
79	Au	Gold	1.04
14	Si	Silicon	1.36

FOV: 269 µm, Mode: 5kV - Map, Detector: BSD Full, Time: OCT 17 2025 12:48

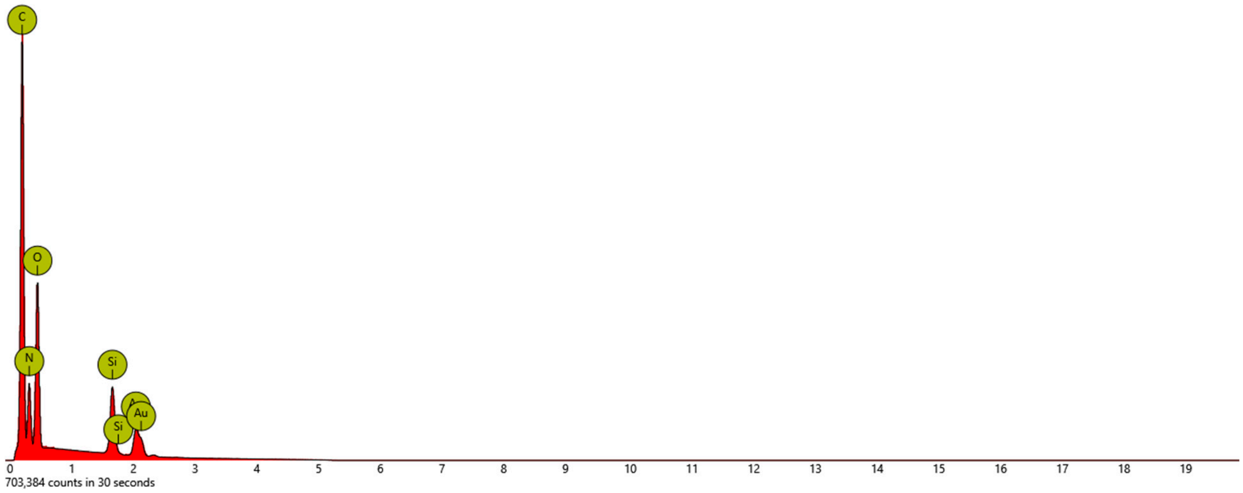

Table 2. Spot

Element Number	Element Symbol	Element Name	Atomic Conc.
6	C	Carbon	60.59
7	N	Nitrogen	20.74
8	O	Oxygen	12.03
79	Au	Gold	2.01
14	Si	Silicon	4.25

FOV: 269 µm, Mode: 5kV - Map, Detector: BSD Full, Time: OCT 17 2025 12:48

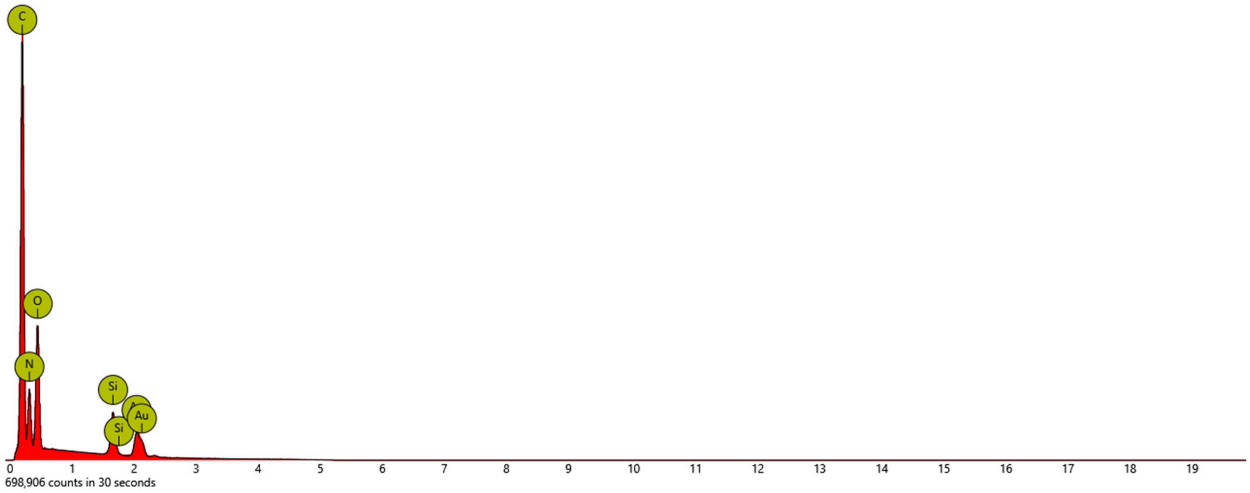

b) PVP/Nb_2O_5

Combined map

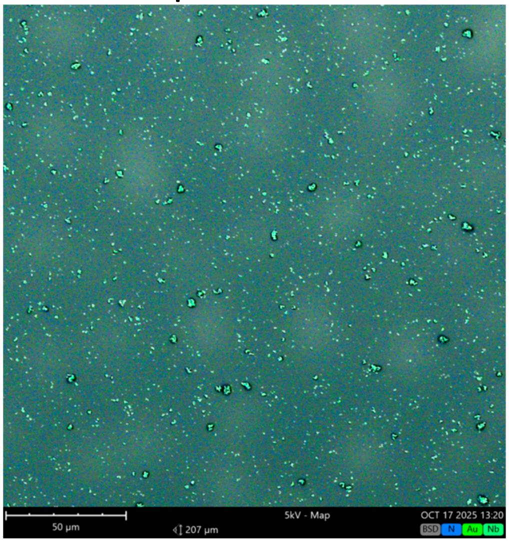

Element Number	Element Symbol	Element Name	Atomic Conc.
6	C	Carbon	43.21
8	O	Oxygen	24.37
14	Si	Silicon	10.89
7	N	Nitrogen	10.62
79	Au	Gold	5.79
41	Nb	Niobium	5.12

Table 3. Spot

FOV: 207 µm, Mode: 5kV - Map, Detector: BSD Full, Time: OCT 17 2025 13:20

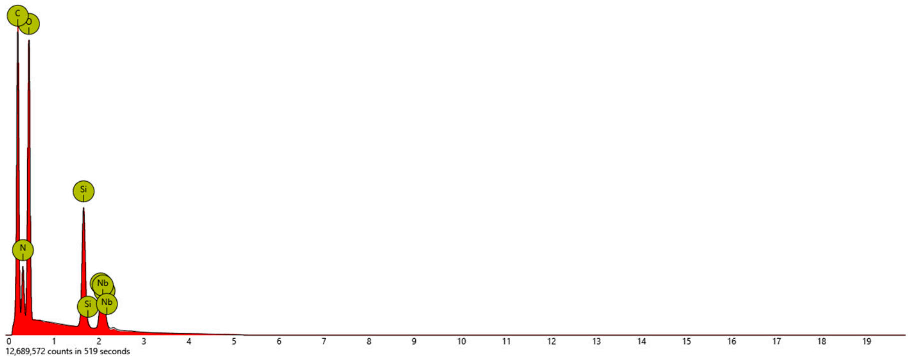

c) Nb_2O_5 cluster

SEM pictures of Nb_2O_5 cluster – zoom 7000x (with determined nanoparticles size – green colour) and zoom 4000x.

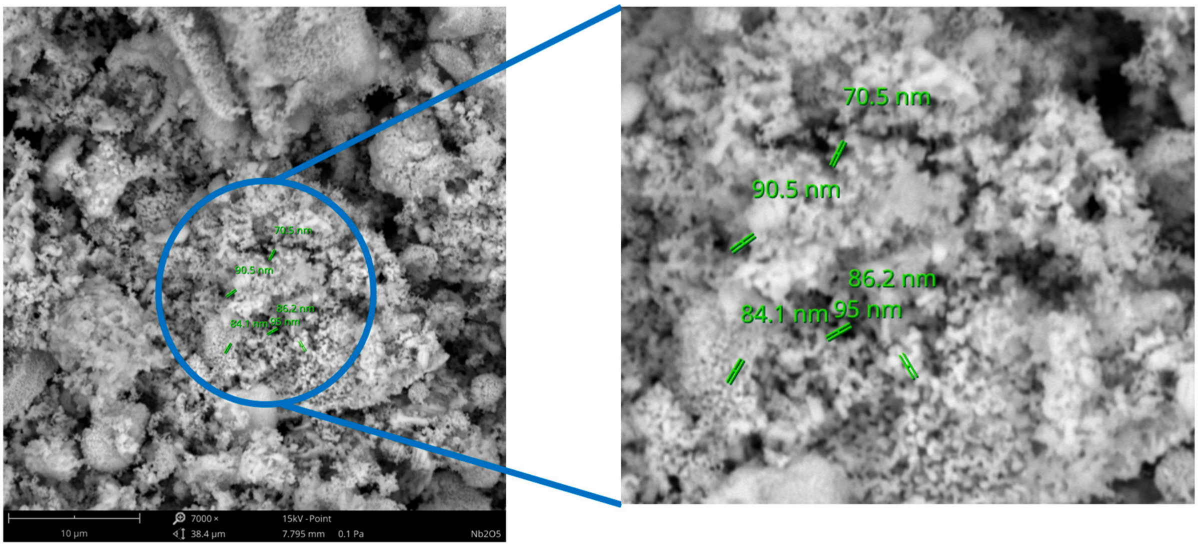

Zoom 7000x

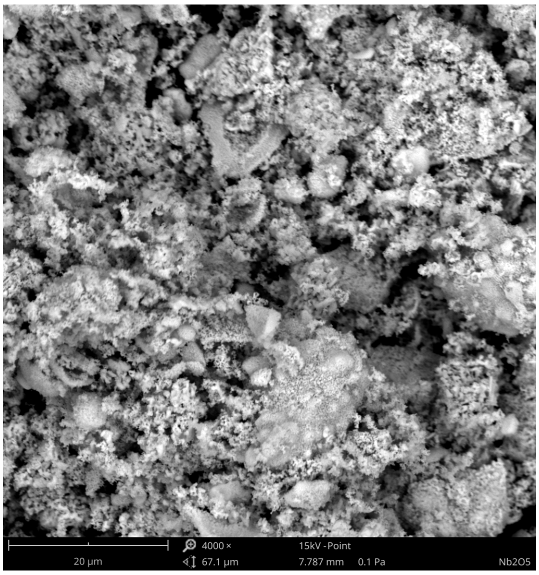

Zoom 4000x

5S. SEM roughness analysis:

For each sample, images were recorded with a field of view of 26.85 µm; spatial filtering was applied with the following parameters: λs= 20 nm (short-wavelength cutoff) and λc = 800 µm (long-wavelength cutoff). 3D height maps were reconstructed, and roughness parameters were calculated: Sa (arithmetical mean height, 3D), Ra (arithmetical mean roughness, 2D), and Rz (maximum height, peak-to-valley), in accordance with ISO 4287/4288 standards. For each composition, at least five independent, randomly selected fields of view were measured on the sample.
In the study, surface roughness parameters were measured for polymer layers: pure PVP and PVP with the addition of Nb_2O_5 at various weight concentrations (5, 15, 25, and 35 wt%). For pure PVP, the measured values were Sa = 90 nm, Ra = 45 nm, and Rz = 148 nm. The addition of Nb_2O_5 caused an increase in the average Sa parameter: 108 nm (5%), 110 nm (15%), 112 nm (25%), and 129 nm (35%) (λs = 20 nm; λc = 800 µm). The increase in roughness with the addition of niobium oxide suggests growing surface irregularity at higher concentrations, resulting from partial particle aggregation and morphological changes, which may also influence the functional properties of the layer.

Table 4. Results of roughness SEM analysis

Sample no.	Sample composition	Sa [nm]	Ra [nm]	Rz [nm]	Topography observations (SEM)
1	PVP (pure)	90	45	148	Relatively smooth surface, homogeneous structure, no visible aggregates.
2	PVP + 5% Nb_2O_5	108	38	133	Slight increase in roughness, possible small particle clusters..
3	PVP + 15% Nb_2O_5	110	54	127	Moderate increase in roughness, slightly granular structure.
4	PVP + 25% Nb_2O_5	112	59	167	More pronounced local irregularities, larger Nb_2O_5 agglomerates appear
5	PVP + 35% Nb_2O_5	129	57	166	Highest roughness among the analyzed samples,

a) PVP

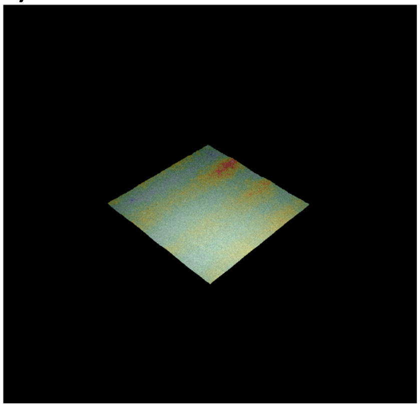

Field of view: 26.85 µm, λs: 20 nm, λc: 800.00 µm, Sa: 90 nm

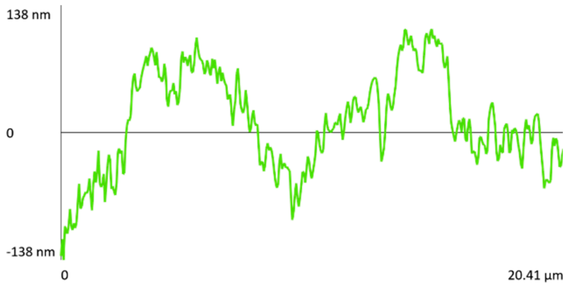

Profile 1 - Rz: 148 nm   Ra: 45 nm

b) PVP/Nb_2O_5 5%

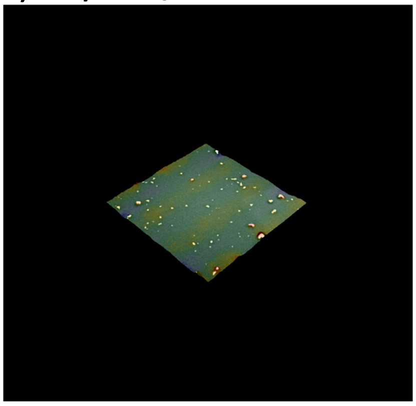

Field of view: 26.85 µm, λs: 20 nm, λc: 800.00 µm, Sa: 108 nm

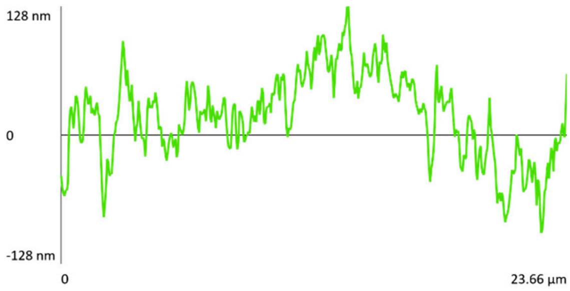

Profile 1 - Rz: 133 nm   Ra: 38 nm

c) **PVP/Nb_2O_5 15%**

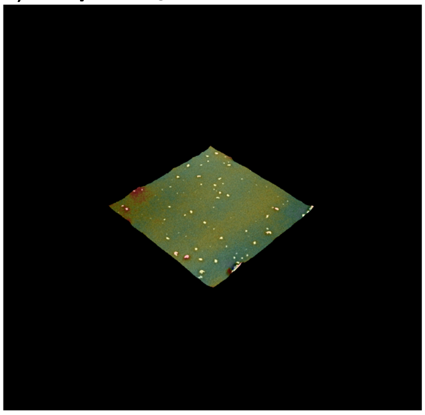

Field of view: 26.85 µm, λs: 20 nm λc: 800.00 µm, Sa: 110 nm

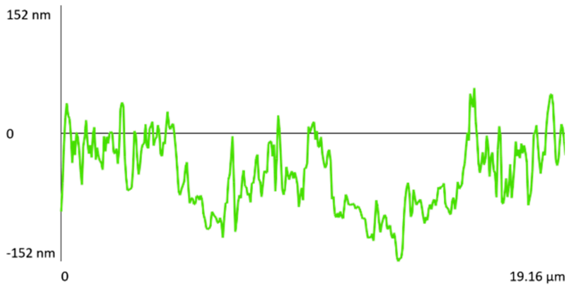

Profile 1 - Rz: 127 nm   Ra: 54 nm

d) PVP/Nb_2O_5 25%

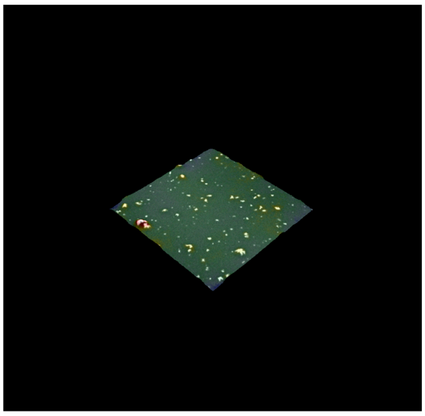

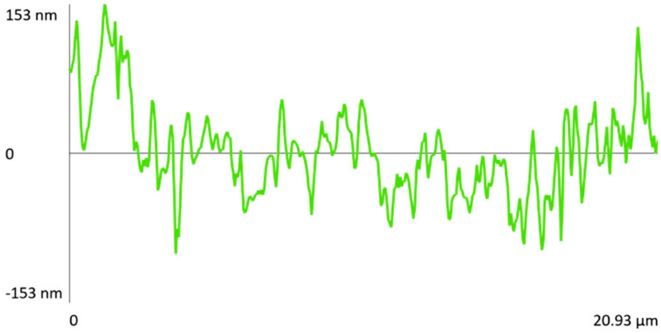

Profile 1 - Rz: 167 nm   Ra: 59 nm

Field of view: 26.85 µm, λs: 20 nm   λc: 800.00 µm
Sa: 112 nm

e) PVP/Nb_2O_5 35%

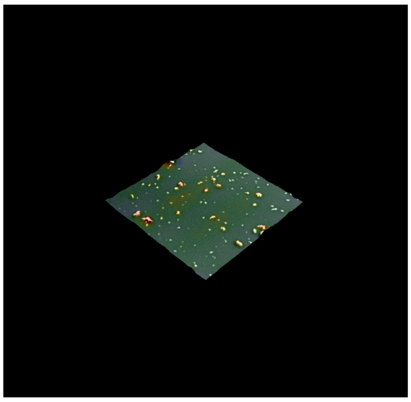

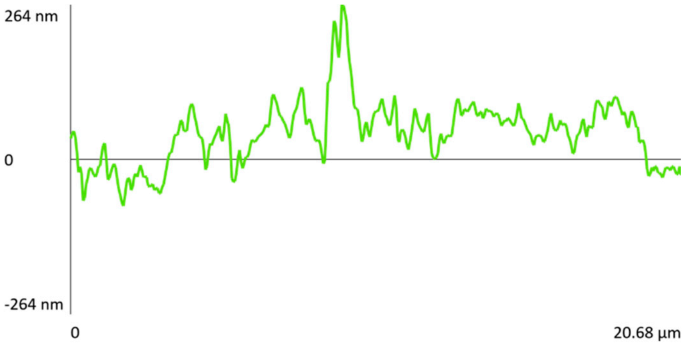

Profile 1 - Rz: 166 nm   Ra: 57 nm

Field of view: 26.85 µm,. λs: 20 nm   λc: 800.00 µm
Sa: 129 nm

f) Cut out of map (resolution: 512x512 pixels)

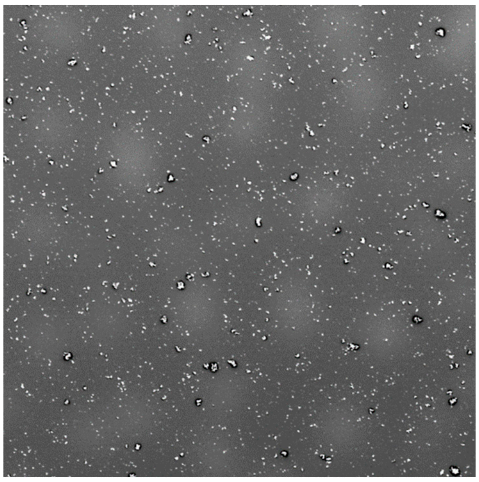

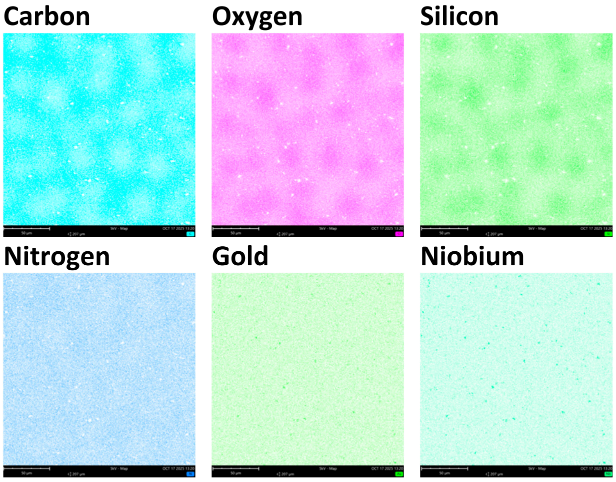

Based on the EDS analysis, six colour maps of elemental distribution were obtained (Carbon, Oxygen, Silicon, Nitrogen, Gold, Niobium). The colours serve an analytical purpose, they enhance the readability of the elemental maps and allow for analysis of phase overlap. Carbon, the main component of the PVP polymer, is evenly distributed across the entire surface of the sample. The intense areas of the C signal confirm the presence and continuity of the organic phase within the composite structure. Oxygen is present in both components of the composite: in the carbonyl groups of PVP and in niobium oxide (Nb_2O_5). Its distribution partially overlaps with the niobium map, indicating the presence of the Nb_2O_5 oxide phase within the polymer matrix. The uniform signal suggests good dispersion of oxide particles in the PVP matrix. The signal from silicon is localized and weak, suggesting that Si is not a structural component of the composite but rather a residue from the substrate. Nitrogen is characteristic of PVP, which contains amide groups. The N map overlaps with carbon-rich areas, confirming the presence of the polymer phase. Its uniform distribution indicates the absence of organic phase segregation. Gold originates from the sputtered layer applied to the sample to improve electrical conductivity during SEM/EDS analysis. Its signal is concentrated on the sample surface and is not related to the actual composite structure. Niobium, derived from Nb_2O_5 particles, shows point-like or finely dispersed distribution, indicating good dispersion of the oxide within the PVP matrix. Areas of higher intensity may suggest the presence of Nb_2O_5 particle agglomerates.

6S. References:

[1s] Gómez Caterine Daza, Rodríguez-Páez Jorge Enrique, The effect of the synthesis conditions on structure and photocatalytic activity of Nb2O5 nanostructures, Processing and Application of Ceramics 12/3 (2018) 218-229, https://doi.org/10.2298/PAC1803218G

[2s] Wang L, Li Y, Han P. Electrospinning preparation of g-C3N4/Nb2O5 nanofibers heterojunction for enhanced photocatalytic degradation of organic pollutants in water. Scientific Reports 11/1 (2021) 22950, doi: 10.1038/s41598-021-02161-x

[3s] L. P. Babu Reddy, H. G. Raj Prakash, Y. T. Ravikiran, Sangappa K. Ganiger, V. Jagadeesha Angadi, Structural and humidity sensing properties of niobium pentoxide-mixed nickel ferrite prepared by mechano-chemical mixing method, Journal of Materials Science: Materials in Electronics, 24 (2020) 21981-21999, 10.1007/s10854-020-04701-z
